# Novel Brain Complexity Measures Based on Information Theory

**DOI:** 10.3390/e20070491

**Published:** 2018-06-25

**Authors:** Ester Bonmati, Anton Bardera, Miquel Feixas, Imma Boada

**Affiliations:** Graphics and Imaging Laboratory, University of Girona, 17003 Girona, Spain

**Keywords:** brain network, complex networks, connectome, information theory, graph theory

## Abstract

Brain networks are widely used models to understand the topology and organization of the brain. These networks can be represented by a graph, where nodes correspond to brain regions and edges to structural or functional connections. Several measures have been proposed to describe the topological features of these networks, but unfortunately, it is still unclear which measures give the best representation of the brain. In this paper, we propose a new set of measures based on information theory. Our approach interprets the brain network as a stochastic process where impulses are modeled as a random walk on the graph nodes. This new interpretation provides a solid theoretical framework from which several global and local measures are derived. Global measures provide quantitative values for the whole brain network characterization and include entropy, mutual information, and erasure mutual information. The latter is a new measure based on mutual information and erasure entropy. On the other hand, local measures are based on different decompositions of the global measures and provide different properties of the nodes. Local measures include entropic surprise, mutual surprise, mutual predictability, and erasure surprise. The proposed approach is evaluated using synthetic model networks and structural and functional human networks at different scales. Results demonstrate that the global measures can characterize new properties of the topology of a brain network and, in addition, for a given number of nodes, an optimal number of edges is found for small-world networks. Local measures show different properties of the nodes such as the uncertainty associated to the node, or the uniqueness of the path that the node belongs. Finally, the consistency of the results across healthy subjects demonstrates the robustness of the proposed measures.

## 1. Introduction

The human brain is a complex system composed of a set of regions, which are segregated in order to perform specific tasks and are also efficiently integrated in order to share information [1]. The mapping of the structure and the functionality of brain networks is therefore a main challenge in understanding the functioning, as it cannot be studied as a group of independent elements. An important first step to understand how the information is shared, is the generation of a comprehensive map. Felleman and Essen [2] represented the connections of different regions of the human brain by defining a connectivity matrix. Later, the idea of a *connectome* [1,3] was introduced, which mapped the neural connections in the brain using networks and graph theory [4,5,6].

In a brain network or graph, nodes correspond to brain regions and edges to structural or functional connections [7,8,9]. To model the brain, different graphs can be used: un-directed binary graphs which are the most popular; weighted graphs that assign weights to the edges according to the degree of connectivity between the nodes; and directed graphs that take the influence of one region in another [7]. Once the graph is built, it needs to be analyzed to describe the hidden information in this dense network.

From the connectome, it has been shown that each brain region has a unique pattern of connections (known as *connectional fingerprint*) [10] that varies across subjects [6] but preserves a similar structure. Different techniques have been applied to describe the topological features of brain networks [11,12,13]. For instance, the independence of large areas, denoted as *integration*, has been studied by the *path length* measure, the *characteristic path length* [14], or the *global efficiency* [15]. The independence of small subsets, defined as *segregation*, can be analyzed by the *clustering coefficient* [14], the *transitivity* [16] or the *modularity* [17]. The importance of individual nodes can be defined with centrality measures such as the *degree* [18], or the *density*. A good summary of these measures can be found in [12].

Global measures have also been proposed to describe the overall network structure of the brain. Studies such as Kennedy et al. [19] suggested that a functional and structural central circuit with different areas acting as a cluster governed the information distribution and integration in the brain. Clusters are densely interconnected areas and are defined as a *rich-club* [20,21,22]. Sporns et al. [23] evidenced small-world properties of human brain networks. Small-world networks are systems with a high level of clusterization, like lattice networks, and with small path lengths, like random graphs.

Information theory has been previously used to study the integration and segregation of brain networks [24,25,26]. For instance, the *neuronal complexity* measure ($C_{N}$) showed a balance between segregation and integration [24,27,28]. Additionally, other measures were proposed such as the *matching complexity* measure ($C_{M}$) that shows the change in $C_{N}$ after receiving signals from the environment [29], the *functional clustering*, which finds groups of regions that are more connected among themselves than with the rest [30], the *degeneracy* measure ($D_{N}$) that describes how structurally different elements are able to perform the same function, and the *redundancy* measure (*R*), that describes how identical elements perform the same function [31,32].

Brain network measures are also able to associate different diseases with disruptions [6,33,34,35]. As an example, Sato et al. [36] used the assessment of the graphs entropy to distinguish subjects with and without hyperactivity [36]. Unfortunatelly, the measures that best describe a brain network are still unknown. Therefore, new network measures showing new properties are required to better understand brain networks and their functioning [37].

In this paper, we use a brain network model, where regions correspond to states of a Markov process, to model impulses as random walks on the brain network [38]. Please note that this model differs from the previous ones [24,27,28], where correlations between subsets are used to study the centrality and segregation. This Markov process-based interpretation provides a solid theoretical framework from which global and local measures can be derived. Global measures provide quantitative values to characterize the whole brain network while local measures, which are based on different decompositions of the global measures, are used to quantify the informativeness associated to each node. To evaluate the proposed measures different synthetic model networks, and structural and functional human networks at different scales are considered.

## 2. Method

### 2.1. Information Theory Basis

Let the alphabet X be a finite set and *X* a random variable taking values *x* in X. The *Shannon entropy* H(X) of a random variable *X* is defined by

(1) $$\begin{array}{c} H\left(X\right)=-\sum_{x\in X}p\left(x\right)\log p\left(x\right), \end{array}$$

where p(x)=Pr[X=x] is the probability of the value *x*. Entropy measures the average uncertainty of a random variable *X*. All logarithms are base 2 and entropy is expressed in bits. In this paper, the convention 0log0=0 is used.

Likewise, let *Y* be a random variable taking values *y* in Y. The *conditional entropy* is defined by

(2) $$H\left(Y|X\right)=-\sum_{x\in X}p\left(x\right)\sum_{y\in Y}p\left(y|x\right)\log p\left(y|x\right),$$

where p(y|x)=Pr[Y=y|X=x] is the conditional probability. The conditional entropy H(Y|X) measures the average uncertainty associated with *Y* if we know the outcome of *X*. In general, H(Y|X)≠H(X|Y), and H(X)≥H(X|Y)≥0.

The *mutual information* (MI) between *X* and *Y* is defined by

(3) $$\begin{array}{cc} I(X;Y)=H(X)-H(X|Y) & =\sum_{x\in X}\sum_{y\in Y}p\left(x,y\right)\log\frac{p(x,y)}{p(x)p(y)}\\ & =\sum_{x\in X}p\left(x\right)\sum_{y\in Y}p\left(y|x\right)\log\frac{p(y|x)}{p(y)}, \end{array}$$

where p(x,y)=Pr[X=x,Y=y] is the joint probability. MI measures the shared information between *X* and *Y*. It can be seen that I(X;Y)=I(Y;X)≥0 [39].

The relative entropy or Kullback-Leibler distance, $D_{KL}\left(p,q\right)$, between two probability distributions *p* and *q*, that are defined over the same alphabet X, is defined by

(4) $$\begin{array}{c} D_{KL}\left(p,q\right)=\sum_{x\in X}p\left(x\right)\log\frac{p(x)}{q(x)}. \end{array}$$

The relative entropy satisfies that $D_{KL}\left(p,q\right)\geq 0$, with equality if and only if p=q. Kullback-Leibler distance is a basic information theory measure to quantify the dissimilarity between two probability distributions, and other measures, like entropy or mutual information, can be reformulated in terms of this.

A *stochastic process* or a discrete-time information source X is an indexed sequence of random variables characterized by the joint probability distribution $p\left(x_{1},x_{2},\ldots ,x_{L}\right)=Pr\left\{\left(X_{1},X_{2},\ldots ,X_{L}\right)=\left(x_{1},x_{2},\ldots ,x_{L}\right)\right\}$ with $\left(x_{1},x_{2},\ldots ,x_{L}\right)\in X^{L}$ for L≥1 [39,40]. The *entropy rate* or entropy density *h* of a stochastic process X is defined by

(5) $$\begin{array}{ccc} h & = & \lim_{L\to \infty }\frac{1}{L}H\left(X_{1},X_{2},\ldots ,X_{L}\right) \end{array}$$

when the limit exists. The entropy rate represents the average information content per symbol in a stochastic process. It is the “uncertainty associated with a given symbol if all the preceding symbols are known” and can be viewed as “the intrinsic *unpredictability*” or “the irreducible *randomness*” associated with the chain [41].

A *stochastic Markov process* [39], or *Markov chain*, is a discrete stochastic process defined over a set of states X which is described by a *transition probability matrix* *P*. In each step, the process makes a transition from its current state *i* to a new state *j* with *transition probability* $P_{ij}=p\left(x_{j}^{t+1}|x_{i}^{t}\right)=Pr\left[X_{t+1}=x_{j}|X_{t}=x_{i}\right]$.

For a *stationary Markov process* (that is, a Markov process whose statistical properties are invariant to a shift in time), the probability of each state *i* converge to a *stationary distribution* $\mu =\{\mu_{1},\ldots ,\mu_{n}\}$ after several steps. The stationary or equilibrium probabilities $\mu_{i}$ fulfill the relation $\mu_{i}=\sum_{j=1}^{n}\mu_{j}P_{ji}$ and also the reciprocity relation $\mu_{i}P_{ij}=\mu_{j}P_{ji}$.

In particular, a Markov process can be considered as a chain of random variables complying with

(6) $$\begin{array}{c} H\left(X_{L}|X_{1},X_{2},\ldots ,X_{L-1}\right)=H\left(X_{L}|X_{L-1}\right). \end{array}$$

An important result is the following theorem: for a stationary Markov chain with stationary distribution $\mu_{i}$, the *entropy rate* or information content is given by

(7) $$\begin{array}{ccc} h & = & H\left(X_{t+1}|X_{t}\right)=-\sum_{i=1}^{n}\mu_{i}\sum_{j=1}^{n}P_{ij}\log P_{ij}, \end{array}$$

where $\mu_{i}$ is the stationary distribution and $P_{ij}$ is the transition probability from state *i* to state *j*.

The *excess entropy* [42,43,44,45] of an infinite chain is defined by

(9) $$\begin{array}{ccc} E & = & \lim_{L\to \infty }\left(H\left(X_{1},X_{2},\ldots ,X_{L}\right)-Lh\right) \end{array}$$

(8) $$\begin{array}{cc} = & \sum_{L=1}^{\infty }\left(H\left(L\right)-h\right), \end{array}$$

where *h* is the entropy rate of the chain, *L* is the length of this chain, and $H\left(L\right)=H\left(X_{L}|X_{L-1},\ldots ,X_{1}\right)$. The excess entropy can be interpreted as the mutual information between two semi-infinite halves of the chain. Another way of viewing this is that excess entropy is a measure of the apparent memory or *structure* in the system, that is, the excess entropy measures how much more random the system would become if we suddenly forgot all information about the left half of the string [46]. For a stationary Markov process, excess entropy coincides with mutual information, and, hence, in this case, mutual information can be seen as a measure of the system structure.

The *erasure entropy* [47] measures the information content of each symbol knowing its context, i.e., the previous and posterior samples. For any stationary process, the erasure entropy is given by

(10) $$\begin{array}{c} H^{-}=\lim_{L\to \infty }H\left(X_{0}|X_{-L}^{-1},X_{1}^{L}\right), \end{array}$$

where $X_{-L}^{-1}$ symbolizes the previous samples (past) and $X_{1}^{L}$ the posterior samples (future).

### 2.2. Markov Process-Based Brain Model

A brain graph can be defined as a pair of sets G=(N,E), where *N* is a brain parcellation of *n* nodes labelled $\left\{N_{1},\ldots ,N_{n}\right\}$, and *E* is a set of *m* edges between two nodes of *N*. This graph can be represented by a connectivity matrix *C* with n×n elements, where $C_{ij}$ gives the connectivity weight between node $x_{i}$ and node $x_{j}$. Please note that for undirected graphs $C_{ij}=C_{ji}$.

In this work, brain functions are modeled as a random walk of a particle on the connectivity graph, where the particle randomly goes from node to node defining a path or a sequence of nodes. From node $x_{i}$, the next node $x_{j}$ is chosen among all nodes connected to node $x_{i}$, with a probability proportional to the weight $C_{ij}$. By introducing this model, we are assuming that the next step in the random walk of a neural impulse is determined only by the region and its connections, but not by previous steps of the random walk.

This model leads to a conditional probability $P_{ij}=p\left(x_{j}^{t+1}|x_{i}^{t}\right)$ given by $C_{ij}/\sum_{i}C_{ij}$. The stationary distribution for this Markov chain assigns probability to node $x_{i}$ proportional to the total weight of the edges emanating from node $x_{i}$ [39]. Thus, the stationary distribution of a node $x_{i}$ is given by

(11) $$\begin{array}{ccc} \mu_{i}=p\left(x_{i}\right) & = & \frac{C_{i}}{C_{T}}, \end{array}$$

where $C_{i}=\sum_{j}C_{ij}$ is the total weight of the edges emanating from node *i* and $C_{T}=\sum_{i}\sum_{j}C_{ij}$ is the sum of the weights of all the edges. Observe that this stationary distribution has an interesting property of locality: it depends only on the total weight and the weight of edges connected to the node and hence, it does not change if the weights in some other part of the graph are changed while keeping constant the total weight.

The definition of this model allows to propose new global and local measures to characterize brain networks. *Global measures* describe by a single value the whole connectivity of the brain, while *local measures* assign a value to each brain region, by considering the contribution of the region to the corresponding global measure. In this work, we propose new measures in three different levels: stationary measures, causal measures, and contextual measures. *Stationary measures* are based on the stationary distribution (i.e., current state). *Causal measures* are based on how the previous states influence the current state in the random walk. Finally, *contextual measures* describe how the context (i.e., the previous and future states) is related to the current state. Table 1 summarizes these measures, which are described in the next subsections.

### 2.3. Global Informativeness Measures

Global measures provide quantitative values to typify the brain connectome as a whole. Depending on which level is considered (stationary, causal, or contextual), three different measures are given: entropy, mutual information, and erasure mutual information.

#### 2.3.1. Entropy

From the stationary distribution μ Equation (11), the Shannon entropy H(μ) Equation (1) measures the average uncertainty of the stationary distribution:

(12) $$\begin{array}{c} H\left(\mu \right)=-\sum_{i=1}^{n}\mu_{i}\log\mu_{i}. \end{array}$$

**Figure 1:**
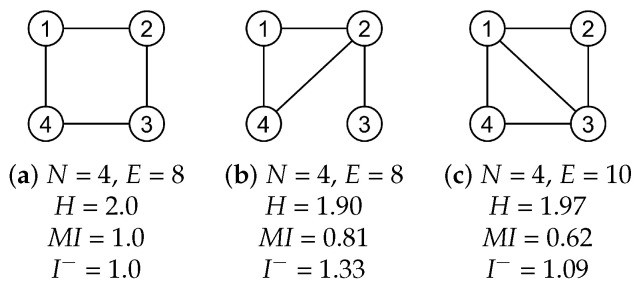
Example values of the entropy (*H*), mutual information (MI) and erasure ($I^{-}$) measures for simple networks (**a**–**c**), where *N* corresponds to the number of nodes and *E* to the number of edges. Networks are weighted and undirected, therefore each edge is counted twice.

Since the probability of each region depends on the weight of their edges, this measure will take high values when all nodes in a network have similar connectivity (weights) and will take low values when there is large variability in terms of number of connections or weights. For instance, in the graph shown in Figure 1a, all nodes have the same number of connections (in this case, each connection has the same weight). Thus, the entropy takes the maximum value given by $\log_{2}N=\log_{2}4=2$, where *N* is the number of nodes. For the graphs of Figure 1b,c, the value of the entropy decreases since the connectivity of the nodes is not equal for all nodes.

#### 2.3.2. Mutual Information

As we have previously mentioned, *mutual information* measures the shared information between two random variables. From our Markov process-based brain model, we propose as a global connectivity measure the mutual information between two consecutive states of the process:

(13) $$\begin{array}{cc} I(X_{t};X_{t+1}) & =H\left(X_{t+1}\right)-H\left(X_{t+1}|X_{t}\right)\\ & =\sum_{x_{i}^{t}\in X}\sum_{x_{j}^{t+1}\in X}p\left(x_{i}^{t},x_{j}^{t+1}\right)\log\frac{p(x_{i}^{t},x_{j}^{t+1})}{p\left(x_{i}^{t}\right)p\left(x_{j}^{t+1}\right)}\\ & =\sum_{i=1}^{n}\mu_{i}\sum_{j=1}^{n}P_{ij}\log\frac{P_{ij}}{\mu_{j}}. \end{array}$$

From Equations (3) and (17), MI can also be seen as the difference between the uncertainty of the states without any knowledge ($H(X_{t+1})$) and the uncertainty of the states when the past is known ($H(X_{t+1}|X_{t})$). In other words, MI measures the information gained when the previous node is known. The higher the MI, the less random the connections. MI can be seen as a measure of brain structure, since it coincides with excess entropy [48].

In the graph of Figure 1a, the fact of knowing the state at a given time *t* (present) leads to the states for the time t+1 (future). For instance, if a given time *t* the random walk is on the state 1, for the next time t+1, the random walk would be either in state 2 or in state 4. Thus, the conditional entropy $H(X_{t+1}|X_{t})$ is $\log_{2}2=1$. The mutual information, given by the entropy (which corresponds to $\log_{2}4=2$) minus the conditional entropy, is also 1. In the other graphs of Figure 1, the conditional entropy is higher, since there are multiple paths, so the uncertainty of the future step is higher. This fact leads to lower values of MI.

#### 2.3.3. Erasure Mutual Information

The idea of the mutual information measure can be extended by considering not only past states, but also future states. Erasure entropy [47] measures the uncertainty of a system when past and future is known. For a Markov process, this measure can be simplified as

(14) $$\begin{array}{ccc} H^{-}\left(X\right) & = & H(X_{t}|X_{t-1},X_{t+1}), \end{array}$$

where X symbolizes the whole process. Please note that, in this case, $X_{t-1}$ symbolizes the past, $X_{t}$ the present, and $X_{t+1}$ the future. From this measure and Equation (3), we can extend mutual information as a measure of the decrease of information when the context (i.e., past and future) is known. Thus, we propose a new global measure, called *erasure mutual information*, defined as

(15) $$\begin{array}{cc} I^{-}\left(X\right) & =I(X_{t};X_{t-1},X_{t+1})\\ & =H\left(X_{t}\right)-H\left(X_{t}|X_{t-1},X_{t+1}\right). \end{array}$$

While the mutual information of Equation (13) measures the loss of information taking into account only the previous node in the random walk (past), erasure mutual information measures the loss of information taking into account the previous node (past) and the next node (future). High values of this measure will show a network with predictable paths to go from node to node, and low values will define a network with several possible paths to go from node to node.

In the graph of Figure 1a, the fact of knowing both past and future states does not reduce the uncertainty of the present (compared with only knowing the past). For instance, if in t−1 the random walk is the state 1 and in time t+1 in 3, the state in *t* can be either 2 or 4. This uncertainty is the same for all possible pairs of past and future states. Thus, the conditional entropy $H(X_{t}|X_{t-1},X_{t+1})$ is $\log_{2}2=1$ and erasure mutual information is also 1. In the other graphs of Figure 1, the knowledge of future and past states, reduces the uncertainty compared with only knowing the past. For instance, in the graph of Figure 1b, if the past state is 4, the present state can be either 1 or 2, but, if the future state is 3, therefore, the present state is, without uncertainty, state 2. Thus, erasure mutual information takes higher values than mutual information.

### 2.4. Local Informativeness Measures

In this section, we describe how global measures can be decomposed in order to characterize the degree of informativeness of each state *i*. When applied to the human connectome, since each state corresponds to an anatomical or functional region, these measures can be seen as the contribution of each node to the whole graph structure, thus, they can describe specific topology of brain areas.

#### 2.4.1. Entropic Surprise

The entropy of *X* can also be interpreted as the expected value of $-\log\left(p(x)\right)$, where *X* is drawn according to probability mass function p(x). Then, in our Markov process-based brain model, the *entropic surprise* value associated to a brain region $x_{i}$ is defined as

(16) $$\begin{array}{ccc} E(x_{i}) & = & -\log\left(\mu_{i}\right), \end{array}$$

where $\mu_{i}$ is the stationary probability of the region $x_{i}$.

This measure uses the stationary probability of a node without taking into account the previous or the next node in the random walk. Nodes with a low value will be nodes with a large number of connections or weights in its connections. Therefore, high values will define nodes with a low number of connections to other brain regions. This measure is inversely proportional to the logarithm of the well known strength measure, which is defined as the sum of the edge weights emanating from the node.

**Figure 2:**
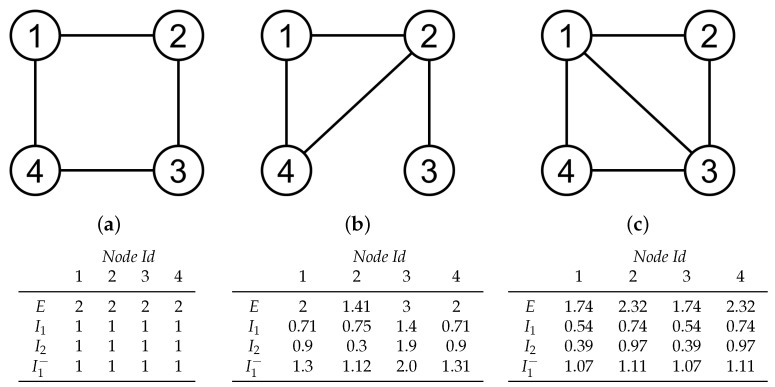
Example values of entropic surprise (*E*), mutual surprise ($I_{1}$), mutual predictability ($I_{2}$) and erasure surprise ($I_{1}^{-}$) measures for simple networks (**a**–**c**). Networks are weighted and undirected, therefore each edge is counted twice.

Some examples are shown in Figure 2. In the left graph, all nodes have the same entropic surprise value, which is given by $-\log_{2}\frac{2}{8}=2$. Please note that each node has 2 edges and there are 4 bidirectional edges (remember that bidirectional edges are counted twice). In the graphs of Figure 2b,c, it can be seen that nodes with high connectivity take lower entropic values.

#### 2.4.2. Mutual Surprise

The interpretation of mutual information explained in Section 2.3 can be extended to define the information associated with a single node $x_{i}\in X$, that is, the information gained on $X_{t+1}$ by knowing the original node $x_{i}$ of the impulse. The definition of *mutual surprise*, denoted by $I_{1}$, can be directly derived from the formula of mutual information Equation (3), taking the contribution of a node $x_{i}$ to $I(X_{t};X_{t+1})$, as follows:

(17) $$\begin{array}{cc} I(X;Y) & =H(X)-H(X|Y)\\ & =\sum_{x\in X}p\left(x\right)\sum_{y\in Y}p\left(y|x\right)\log\frac{p(y|x)}{p(y)},\\ & =\sum_{x\in X}p\left(x\right)I_{1}\left(x;Y\right). \end{array}$$

Mutual surprise was used by DeWeese and Meister [49] to emphasize the fact that the observation of *x* has moved the estimate of another variable *Y* towards values that seemed very unlikely prior to the observation. $I_{1}$ always takes positive values and it can be shown that $I_{1}$ is the only positive decomposition of MI [49].

We reformulate Equation (17) in the framework of the Markov process as follows:

(18) $$\begin{array}{cc} I(X_{t};X_{t+1}) & =\sum_{x_{i}^{t}\in X}p\left(x_{i}^{t}\right)\sum_{x_{j}^{t+1}\in X}p\left(x_{j}^{t+1}|x_{i}^{t}\right)\log\frac{p(x_{j}^{t+1}|x_{i}^{t})}{p(x_{j}^{t+1})}\\ & =\sum_{x_{i}^{t}\in X}p\left(x_{i}^{t}\right)I_{1}\left(x_{i}^{t};X_{t+1}\right), \end{array}$$

where

(19) $$\begin{array}{cc} I_{1}\left(x_{i}^{t};X_{t+1}\right) & =\sum_{x_{j}^{t+1}\in X}p\left(x_{j}^{t+1}|x_{i}^{t}\right)\log\frac{p(x_{j}^{t+1}|x_{i}^{t})}{p(x_{j}^{t+1})}\\ & =\sum_{j=1}^{n}P_{ij}\log\frac{P_{ij}}{\mu_{j}} \end{array}$$

expresses the surprise about $X_{t+1}$ from observing $x_{i}^{t}$, i.e., how “surprising” are the nodes connected with the original node. Observe that surprise $I_{1}\left(x_{i}^{t};X_{t+1}\right)$ is high when $p(X_{t+1}|x_{i}^{t})$ is very different from $p(X_{t+1})$ (i.e., the stationary distribution), thus, the region $x_{i}$ is connected with regions which are less connected considering all the connections.

$I_{1}$ can be seen as the Kullback-Leibler distance see Equation (4) between $p(X_{t+1})$ (i.e., the stationary distribution) and $p(X_{t+1}|x_{i}^{t})$ (i.e., the distribution of future states if, in the present state, the random walk is on node $x_{i}$). Thus, those nodes that are connected with more likely nodes (in terms of the stationary distribution) will lead to low values of $I_{1}$, while those with very specific connections or connected with few unlikely nodes will have high $I_{1}$ values. This can be seen, for instance, in node 3 of graph shown in Figure 2b.

#### 2.4.3. Mutual Predictability

DeWeese and Meister [49] defined the specific information $I_{2}$, which we call *mutual predictability*, using another decomposition of mutual information obtained from Equation (3):

(20) $$\begin{array}{cc} I(X;Y) & =H(Y)-H(Y|X)\\ & =\sum_{x\in X}p\left(x\right)H\left(Y\right)-\sum_{x\in X}p\left(x\right)H\left(Y|x\right)\\ & =\sum_{x\in X}p\left(x\right)I_{2}\left(x;Y\right), \end{array}$$

where

(21) $$\begin{array}{cc} I_{2}\left(x;Y\right) & =H(Y)-H(Y|x)\\ & =-\sum_{y\in Y}p\left(y\right)\log p\left(y\right)+\sum_{y\in Y}p\left(y|x\right)\log p\left(y|x\right) \end{array}$$

expresses the change in uncertainty of *Y* when *x* is observed. In our case, we reformulate $I_{2}$ in the framework of the Markov process as follows

(22) $$\begin{array}{cc} I_{2}\left(x_{i}^{t};X_{t+1}\right) & =H\left(X_{t+1}\right)-H\left(X_{t+1}|x_{i}^{t}\right)\\ & =-\sum_{x_{j}^{t+1}\in X}p\left(x_{j}^{t+1}\right)\log p\left(x_{j}^{t+1}\right)+\sum_{x_{j}^{t+1}\in X}p\left(x_{j}^{t+1}|x_{i}^{t}\right)\log p\left(x_{j}^{t+1}|x_{i}^{t}\right)\\ & =H\left(\mu \right)+\sum_{j=1}^{n}P_{ij}\log P_{ij}. \end{array}$$

Observe that this measure expresses the difference between global entropy of the graph (i.e., the entropy of the stationary distribution) and entropy of future states of the random walk from node $x_{i}$. So, this comparison is done globally and, contrarily to the $I_{1}$ measure, it is not affected by the stationary probability of the nodes that is connected to. Another property that fulfills $I_{2}$ is additivity, i.e., the information obtained about *X* from two observations, y∈Y and z∈Z, is equal to that obtained from *y* plus that obtained from *z* when *y* is known. Additivity is a desirable property that responds to the intuitive notion that information accumulates additively over a sequence of observations. Because of the additivity property, DeWeese and Meister [49] prefer $I_{2}$ against $I_{1}$.

Please note that $I_{2}\left(x_{i}^{t};X_{t+1}\right)$ can take negative values. In this case, this means that a certain region $x_{i}$ is connected with more uncertainty than the mean connectivity of the whole brain. Regions with high values of $I_{2}$ (like node 3 in the graph of Figure 2b) greatly reduce the uncertainty in $X_{t+1}$ and, thus, they are very significant in the relationship between two consecutive steps in the random walk, $X_{t}$ and $X_{t+1}$. Regions with low values of $I_{2}$ (like node 2 in the graph of Figure 2b) are assumed to be broadly connected with other brain regions. From this interpretation, we can say that $I_{2}$ expresses the capacity of prediction for a given brain region.

#### 2.4.4. Erasure Surprise

In this section, we propose a novel measure based on the decomposition of the erasure mutual information Equation (15) measure. Remember that erasure mutual information represents the reduction of uncertainty when the context (i.e., both past and future) is known.

Then, we can decompose the erasure mutual information measure as:

(23) $$\begin{array}{cc} I^{-}\left(X\right) & =H\left(X_{t}\right)-H\left(X_{t}|X_{t-1},X_{t+1}\right)\\ & =\sum_{x_{i}^{t}\in X}p\left(x_{i}^{t}\right)I_{1}^{-}\left(x_{i}^{t};X\right), \end{array}$$

where

(24) $$\begin{array}{ccc} I_{1}^{-}\left(x_{i}^{t};X\right) & = & \sum_{x_{j}^{t-1}\in X}\sum_{x_{k}^{t+1}\in X}p\left(x_{j}^{t-1},x_{k}^{t+1}|x_{i}^{t}\right)\log\frac{p(x_{j}^{t-1},x_{k}^{t+1}|x_{i}^{t})}{p(x_{j}^{t-1},x_{k}^{t+1})} \end{array}$$

(25) $$\begin{array}{cc} = & \sum_{j=1}^{n}\mu_{j}P_{ji}\sum_{k=1}^{n}\frac{P_{ik}}{\mu_{i}}\log\frac{P_{ji}P_{ik}}{\mu_{i}Q_{jk}} \end{array}$$

and $Q_{jk}=\sum_{i=1}^{n}P_{ji}P_{ik}$. $I_{1}^{-}$ is the *erasure surprise* associated to the region $x_{i}$ and it always takes positive values. Observe that $I_{1}^{-}$ can be seen as the Kullback-Leibler distance see Equation (4) between $p(X_{t-1},X_{t+1})$ (i.e., joint probability of being at t−1 on node $x_{j}$ and at t+1 on node *k*) and $p(X_{t-1},X_{t+1}|x_{i}^{t})$ (i.e., the same as the latter but conditioned to the fact that at *t* the random walk is on node $x_{i}$. Thus, those nodes that connect brain regions which are already connected will lead to low values of $I_{1}^{-}$, and are likely to belong to the same cluster. This can be seen, for instance, in node 2 of the graph shown in Figure 2b. Instead, nodes that connect nodes which would not be connected otherwise (unique paths), will have high values (node 3 of graph shown in Figure 2b).

## 3. Material

### 3.1. Synthetic Network Models

The human connectome has been defined as a network with an average short path length which gives a high efficiency in transferring information, a high clustering which provides robustness to random errors, a degree distribution similar to networks with hubs, and a modular community structure [18]. According to these properties, random, lattice, and small-world networks are models that can represent the human connectome. If efficiency was the only property used in the network design, the network would be random [18], with low clustering, short path length [50], and all connections equally probable. However, it is clear that the cortex is not just a uniform system of random connected neurons since random graphs cannot encode and process information [19]. If wiring cost was the priority, the network would be similar to a lattice graph with long paths and high clustering. If we aim for a balance between high clustering and average short path length, then small-world networks are the more accurate representation for both structural and functional networks. For this reason, to illustrate features of the proposed measures, we created three datasets containing random, lattice, ring lattice, and small-world networks.

The first dataset contained random, lattice, ring lattice, and small-world networks with 128 nodes and different number of edges ranging from 128 to 8192 with a step of 128 edges. The second dataset contained the same network models with 256 nodes and edges ranging from 256 to 8192 with a step of 128 edges. Please note that these two datasets provide equivalent networks but with different densities, since the number of nodes was fixed and the number of edges varied. Additionally, a third dataset was created with nodes ranging from 32 to 512 with a step of 32 and a fixed density of 0.4 (varied number of edges). For all graphs, a random weight ranging from 0 to 1 was assigned to all the edges.

**Figure 3:**
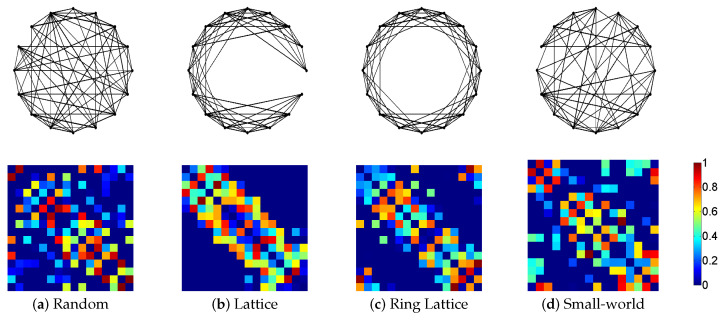
Example of network models (synthetic dataset) used in this work. Each model has the corresponding connectivity matrix illustrated at the bottom. (**a**) Non directed random network (16 nodes, 120 edges); (**b**) Non directed lattice network (16 nodes, 118 edges); (**c**) Non directed ring lattice network (16 nodes, 122 edges); (**d**) Non directed small-world network (16 nodes, 116 edges and cluster size 2).

The network models were created using the Brain Connectivity Toolbox (BCT) [12]. This toolbox contains a large selection of reference network models and measures that have been previously used in several studies [51,52,53]. To create the undirected random networks, we used the function makerandCIJ_und which generates graphs with no connections on the diagonal (see Figure 3a). The directed non-ring lattice networks were created with the function makelatticeCIJ. This lattice is made by placing connections as close as possible to the main diagonal, without wrapping around, and with no connections on the diagonal (see Figure 3b). The ring directed lattice networks were created with the function makeringlatticeCIJ. In this case, the lattice is also made by placing connections close to the diagonal, but wrapping around (see Figure 3c). Finally, directed small-world networks were created with the function makeevenCIJ. These networks have a specific number of fully connected nodes linked together but with a balanced random connections (see Figure 3d). To transform directed graphs to undirected graphs, all values above diagonal were copied below the diagonal, therefore, all synthetic networks used in this work are weighted and undirected.

### 3.2. Human Datasets

Human datasets were used to test the proposed measures with real data. To show the applicability of our method, we considered both functional and structural brain networks.

#### 3.2.1. Anatomic Dataset

**Figure 4:**
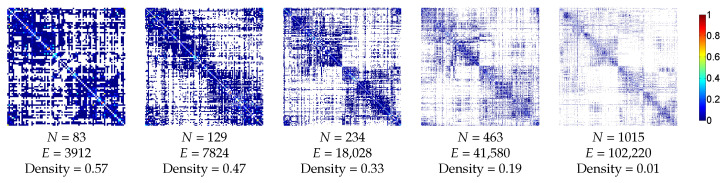
Illustration of the averaged structural connectivity matrices of the anatomic dataset with the corresponding number of nodes (*N*), edges (*E*) and density. 0 values are represented in white. Edges were resorted to place more edges closer to the diagonal for visualization purposes only.

To study the human structural network, we used normalized connection matrices created from MRI tractography [54]. The connectivity matrices were from 10 different subjects at 5 different scales, corresponding to 83, 129, 234, 463 and 1015 cortical and subcortical ROIs. Subjects were all males aged 22 ± 1.3 years old. Edge weights were given by the connectivity density which corresponds to the number of fibers divided by the average of the region surface and by the average length of the fibers. All values were positive, and values on the diagonal were eliminated. The average matrices of the 10 patients for each scale were also created. Figure 4 shows the averaged matrices for the 5 different scales. Edges were resorted to place more edges closer to the diagonal for visualization purposes only.

#### 3.2.2. Functional Dataset

**Figure 5:**
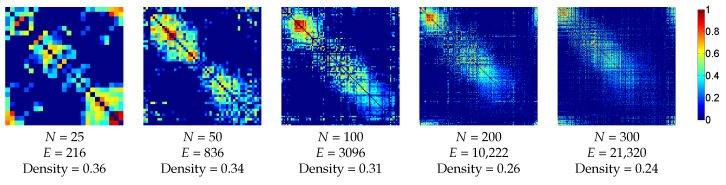
Illustration of the averaged functional connectivity matrices of the functional dataset with the corresponding number of nodes (*N*), edges (*E*) and density.

Independent component analysis (ICA) is a widely used method to generate functional brain networks of the brain during rest and task. For our analysis, we used the HCP500-PTN functional dataset which belongs to the Human Connectome Project (HCP) beta-release of group-ICA maps [55,56,57]. This dataset contains functional network matrices of 461 subjects at 5 different scales (25, 50, 100, 200 and 300). For our experiment, we used the approach where the principal eigen-timeseries are estimated and a full normalized temporal correlation has been used. The original matrices contain positive and negative values and no values on the diagonal, but for our experiments, the matrices were thresholded (Z>5) and the negative values were eliminated. The averaged networks were also used. Figure 5 shows the averaged functional matrices at different scales.

### 3.3. Standard Network Measures

The BCT toolbox [12] provides different complex network measures to describe either structural or functional brain connectivity. To evaluate the proposed approach we compared our measures with standard measures included in the BCT. The clustering coefficient is a measure of segregation and expresses the fraction of triangles around a node. The node eccentricity is a measure of distance defined as the maximal shortest path length between a node and any other node. Finally, the node strength is a measure of similarity defined as the sum of weights of links connected to the node.

## 4. Results and Discussion

In this section, we apply the proposed measures to the synthetic network models and to the human structural and functional connectomes. The results with the global measures and local measures are shown and a comparison with standard measures is presented.

### 4.1. Global Measures

Firstly, to show the behavior of the global measures, we characterize the synthetic network models (random, lattice, ring lattice and small-world) from the first and second dataset defined in Section 3.1, with 128 and 256 nodes. We apply the global measures (entropy, mutual information, and erasure mutual information) which give a single value per graph.

**Figure 6:**
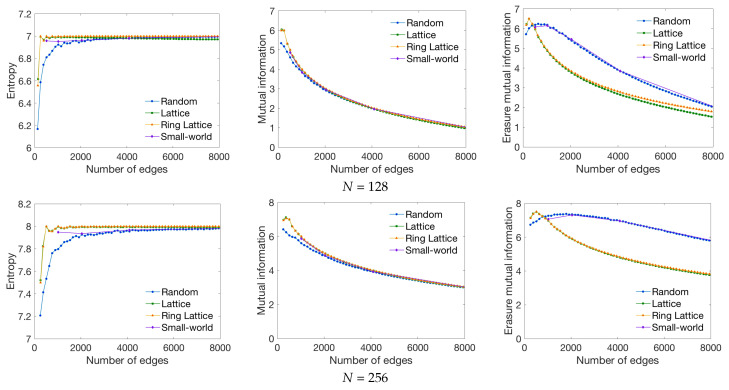
Behavior of the entropy, mutual information and erasure mutual information measures for each network model when the number of edges is increased (from 0 to 8192) and the number of nodes (*N*) is kept constant (128 nodes on top row and 256 nodes on bottom row).

The first column of Figure 6 presents the entropy measure results. Observe that, when the number of edges increases, the entropy measure tends to a constant value for all types of graphs. This is due to the fact that the higher the number of edges, the more similar the node probability. Thus, the entropy tends to $\log_{2}N$, where *N* is the number of nodes (i.e., for 128 nodes the entropy tends to 7 and for 256 nodes to 8). The slightly decreasing tendency of high values in lattice networks is due to the boundary conditions of extreme nodes which have a lower number of connections which leads to an entropy drop.

The second column of Figure 6 shows the behavior of the mutual information measure. In this case, when the number of edges increases, the mutual information of the graph decreases for all types of networks. This is due to the fact that the higher the number of connections, the lower the correlation between consecutive states. For a very low number of edges, we can see that first, the mutual information increases and then decreases. This is due to the fact that for low densities, there are nodes not connected with any node, leading to a decrease of the overall mutual information. Since different tracking methods may provide different number of fibers for a given parcellation [58], the optimal point found with the mutual information measure may allow to find the minimum number of fibers needed for a given brain parcellation to study ring lattice and lattice properties. For a low number of edges, we can also observe that lattice and ring lattice graphs have a slightly higher mutual information than random and small-world graphs. This is due to the higher degree of structure of these kind of graphs, which is what the mutual information measure quantifies.

The third column of Figure 6 presents the values of the erasure mutual information measure. In this case, when the number of edges increases, for all networks, the measure tends to decrease. Note that when there are only a few edges, the uncertainty when past and future states are known is very low ($H(X_{t}|X_{t-1},X_{t+1})$, the second term of Equation (15)), leading to high $I^{-}\left(X\right)$ values. When the number of edges increases, the uncertainty tends to increase, thus, the $I^{-}\left(X\right)$ tends to decrease. For this measure, different behaviors can be observed depending on the graph type. For instance, the lattice and ring lattice graphs have a lower erasure mutual information compared to random and small-world graphs. This is due to the fact that the erasure mutual information takes into account the previous node and next node, and for lattice networks, nodes tend to be connected with the closest ones, thus, globally there is more uncertainty. An interesting behavior can be observed for the random and small-world graphs where the measure reaches an optimal point with a larger number of connections compared to lattice and ring lattice networks. In this case, for a low number of edges, there are nodes which are not connected or only connected with intra-module nodes. Thus, all the paths are within the same module. When the number of edges slightly increases, there are more paths that connect different modules but the probability of these paths is very low. Therefore, the erasure mutual information slightly increases. After the optimal point, the erasure mutual information decreases due to the larger number of connections between different modules that increase the uncertainty.

**Figure 7:**
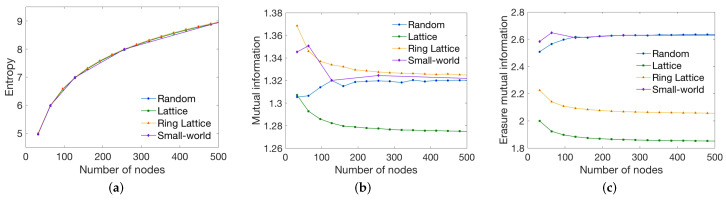
Behavior of the (**a**) entropy; (**b**) mutual information; (**c**) and erasure mutual information measures when the number of nodes is increased (from 0 to 500) while the density is kept constant to 0.4.

Secondly, we generated different graphs, in this case, modifying the number of nodes but preserving the density (number of edges divided by the number of edges of the complete graph) to 0.4, which is the third dataset described in Section 3.1. Figure 7 shows the behavior of the global measures when the number of nodes increases. As it can be seen, the entropy value increases with the logarithm of the size for a constant edge density. This is consistent with the results of the first experiment where entropy tends to $\log_{2}N$, being *N* the number of nodes. On the contrary, mutual information is not very sensitive for random networks since its connections are randomly placed, so fixing the graph density, the structure of the networks remains similar. A comparable behavior can be observed for the small-world networks. In this case, graphs with a low number of nodes, have a higher mutual information due to more intra-module connections, and, as a consequence, if we increase the number of nodes, the number of edges also increases. On the other side, we can observe that while ring lattice network have a high value, lattice network have a very small value. This is because two nodes of the lattice network are not connected and, the rest of the nodes, have a higher degree compared to ring lattice. Consequently, there are less unique paths. Finally, erasure mutual information is not very sensitive to the graph size but to the graph topology. Random and small world have higher values compared to ring lattice and lattice. This is due to the existence of a large amount of connecting paths for neighbor nodes in ring and lattice networks, so paths are not unique. Since for lattice graphs two nodes are not connected, the rest of the nodes have a slightly higher degree, and, as a result the overall predictability is lower. If we increase the number of nodes we have to increase also the number of edges, thus, as a result, the degree of the nodes increases. Because of this, if we focus on the values for a low number of nodes, we can observe that the erasure mutual information for lattice and ring lattice slightly decreases, and, for random and small-world, increases. The erasure mutual information measure takes into account the next node but also the previous one. Therefore, increasing the degree in the ring lattice and the lattice networks, the overall uncertainty increases. On the contrary, for random and small-world networks with a low node degree, paths are more unique for a low number of nodes. Increasing the number of nodes while keeping the density the same, the erasure mutual information tends to stabilize.

**Figure 8:**
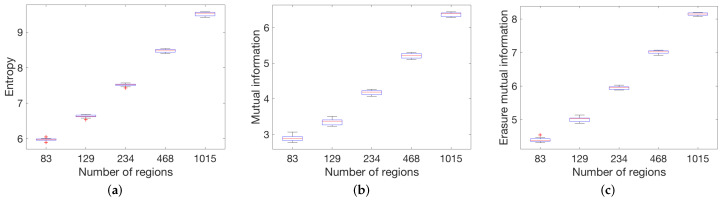
Box-plots showing median, 25th and 75th percentiles for global measures ((**a**) entropy; (**b**) mutual information; (**c**) and erasure mutual information) when applied to the 10 structural connectomes with 83, 129, 254, 463 and 1015 partitions.

**Figure 9:**
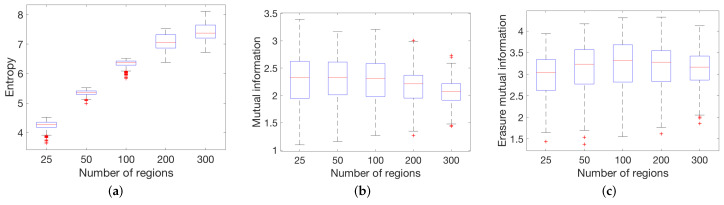
Box-plots showing median, 25th and 75th percentiles for global measures ((**a**) entropy; (**b**) mutual information; (**c**) and erasure mutual information) when applied to 463 functional connectomes with 25, 50, 100, 200 and 300 partitions.

To evaluate the global measures with anatomical data, we applied the global measures to the anatomic and functional datasets at different scales described in Section 3.2. Figure 8 shows the result of entropy, mutual information, and erasure mutual information for the 10 structural networks with 83, 129, 234 and 1015 partitions. Observe that all measures have a similar behavior for all the patients which demonstrates that the measures are consistent among all patients. Figure 9 shows the result of the global measures applied to 468 functional networks with 25, 50, 100, 200 and 300 nodes. In this case, the entropy measure has the same behavior as the structural network. Moreover, since the density is similar between different partitions, the mutual information and the erasure measures have a more uniform value. The same effect has been shown in the behavior of the mutual information and the erasure for model graphs with a constant density.

### 4.2. Local Measures

In this section, we compare local measures (entropic surprise, mutual surprise, mutual predictability and erasure surprise) with standard measures provided in the BCT. In addition, we show the result of the measures applied to the structural and functional human connectivity networks.

**Figure 10:**
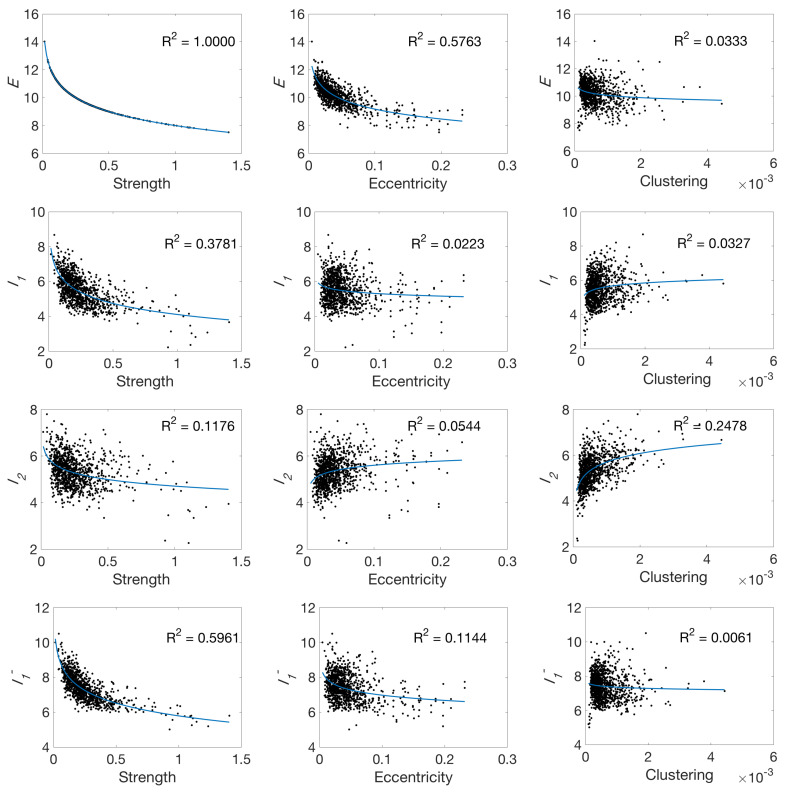
Relationship between the proposed local measures (entropic surprise (*E*), mutual surprise ($I_{1}$), mutual predictability ($I_{2}$) and erasure surprise ($I_{1}^{-}$) and standard measures (strength, eccentricity and clustering) using the structural averaged connectivity matrix network with 1015 nodes.

First of all, we provide a comparison of the proposed local measures with *strength*, *eccentricity* and *clustering* measures. Strength measures the sum of the weights for each node, eccentricity the maximal shortest path length between a node and any other node, and clustering the fraction of triangles in the node. To carry out this experiment, we have considered the averaged connectivity matrix created from the 10 structural networks with 1015 nodes of the anatomic dataset described in Section 3.2.1. The purpose of this experiment is to demonstrate the feasibility and application of the method in a real clinical scenario. Results are shown in Figure 10. From left to right, columns represent entropic surprise (*E*), mutual surprise ($I_{1}$), mutual predictability ($I_{2}$), and erasure surprise ($I_{1}^{-}$), and, from top to down, rows represent the value of our measure with respect to strength, eccentricity, and clustering, respectively. For each scatter plot, *x*-axis represents the standard measure value and the *y*-axis the value of our measure. In each plot, the logarithmic curve ($f\left(x\right)=a+b\log_{2}\left(x\right)$) that best fits to the data and the determination coefficient, $R^{2}$, of the data model are also shown. We can see that the surprise measure is directly related to the strength since both depend on the weight of the node and the surprise measure is mathematically defined as minus the logarithm of the strength see Equation (16). As it can be seen, the other measures are moderately correlated (mainly $I_{1}$ and $I_{1}^{-}$) to the strength. This is not directly related to their mathematical definition, but by the fact that those nodes with more connections (high strength) tend to have more uncertainty on their connections and, thus, lower measure values. Comparing with the eccentricity measure, we can observe that nodes with a high maximal shortest path length (high eccentricity) tend to not be highly connected (low *E* value). On the other side, nodes with a low eccentricity are highly connected. The other measures do not demonstrate significant correlation with eccentricity. With respect to the clustering measure, $I_{2}$ is the only measure that slightly correlates with it. This can be explained by the fact that those nodes with a high clustering coefficient will tend to have less uncertainty on their connections.

**Figure 11:**
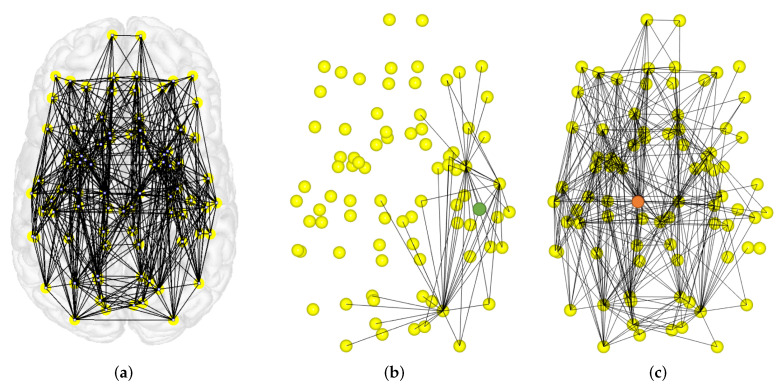
(**a**) Illustration of all the connections in the structural dataset; (**b**) Right hemisphere transverse temporal region (green) connections including its neighbors connections; (**c**) Left hemisphere thalamus proper (orange) connections including its neighbors connections. This figure has been generated using the VisualConnectome software [59].

Finally, we show the value of each measure for each node of the human structural and functional averaged networks, with 83 and 25 partitions, respectively. Figure 11a shows all the nodes for the structural network in yellow and the connections between nodes in black.

**Figure 12:**
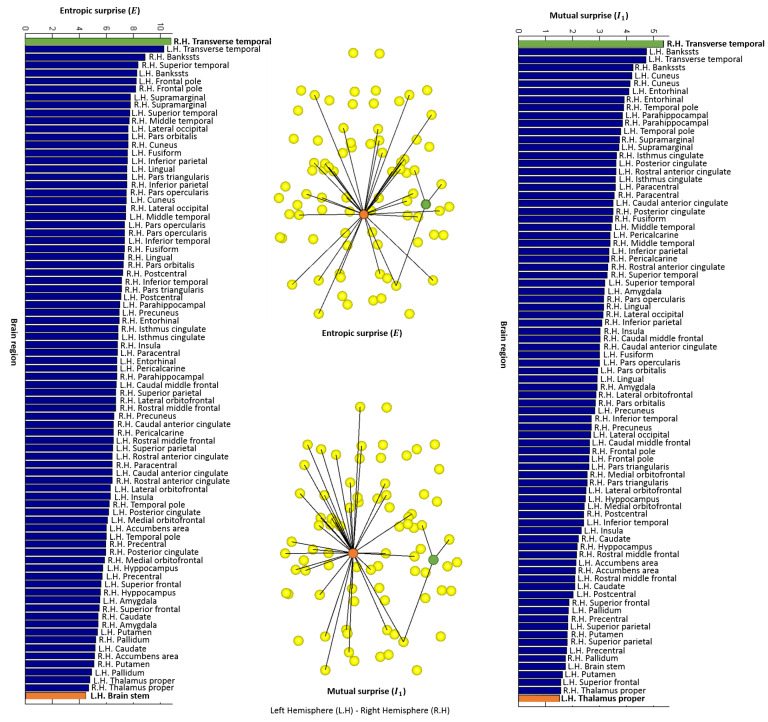
On the left, entropic surprise values obtained with the averaged structural network with 83 partitions. The maximum and minimum values have been represented on the brain network (first image of the central column). The green node corresponds to the right hemisphere transverse temporal area and the orange to the brain stem. On the right, mutual surprise values obtained with the same network. The maximum and minimum values have been represented on the brain network (second image of the central column). The green node corresponds to the right hemisphere transverse temporal area and the orange to the thalamus proper.

The value of the entropic surprise *E* for each node of the human structural averaged network, with 83 partitions is shown on the left histogram of Figure 12. This measure is equivalent to the classic strength measure, where nodes with high values are nodes not highly connected or with low weights, which lead to a low stationary probability. The maximum and minimum *E* values corresponds to the right hemisphere transverse temporal and the brain stem, respectively. These nodes have been represented on the brain network in green and orange (see Figure 12 top image of the central column). The value of the mutual surprise $I_{1}$ for each node is shown on the right histogram of Figure 12. High values correspond to nodes connected to poorly connected nodes (nodes with a low number of connections), while low values correspond to nodes connected to highly connected nodes. This fact is illustrated on the bottom image of the central column of Figure 12 where the right hemisphere transverse temporal, represented as a green node, has the maximum value and the left hemisphere thalamus proper, represented as an orange node, has the minimum one. Comparing entropic surprise and mutual surprise for the structural connectome, we observe an organization, where nodes highly connected are also nodes connected to similar nodes in terms of probability, and nodes not highly connected are connected to nodes with a very different probability compared to them.

**Figure 13:**
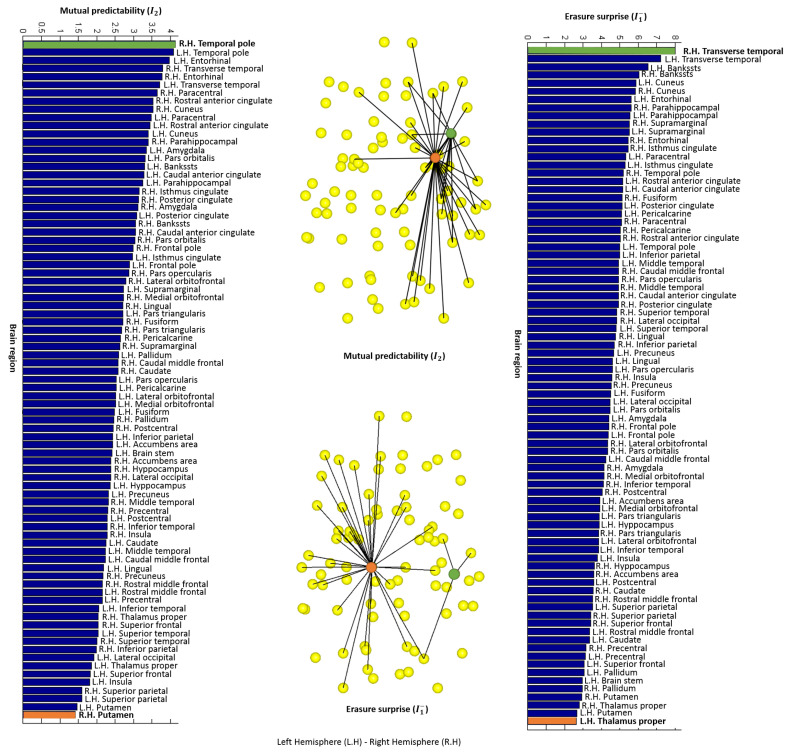
On the left, mutual predictability values obtained with the averaged structural network with 83 partitions. The maximum and minimum values have been represented on the brain network (first image of the central column). The green node corresponds to the right hemisphere temporal pole area and the orange to the putamen. On the right, erasure surprise values obtained with the same network. The maximum and minimum values have been represented on the brain network (second image of the central column). The green node corresponds to the right hemisphere transverse temporal area and the orange to the thalamus proper.

The value of the mutual predictability $I_{2}$ for each node of the human structural averaged network, with 83 partitions is shown on left histogram of Figure 13. Remember that, for nodes with a high mutual predictability, the distribution of connections with other nodes have a low entropy. For instance, observe the first image of the central column in Figure 13, the green node, which corresponds to the right hemisphere temporal pole, has the highest $I_{2}$ value. On the other hand, nodes with low values have more uncertainty in predicting the next node. In this case, the lowest $I_{2}$ value corresponds to the right hemisphere putamen, represented as an orange node. The value of the erasure surprise $I_{1}^{-}$ is shown on the right histogram of Figure 13. Nodes with high values are nodes that connect different areas otherwise not connected or less connected, like a bridge or a hub. For example, the right hemisphere transverse temporal, shown in Figure 11b together with its neighbor connections, is the region with a higher value in the bottom image of the central column in Figure 13. On the other side, nodes with low values, are nodes that belong to a cluster since there are multiple paths connecting its neighbors. In this case, the lower value of the histogram corresponds to the left hemisphere thalamus proper, which is shown in Figure 11c together with its neighbor connections.

**Figure 14:**
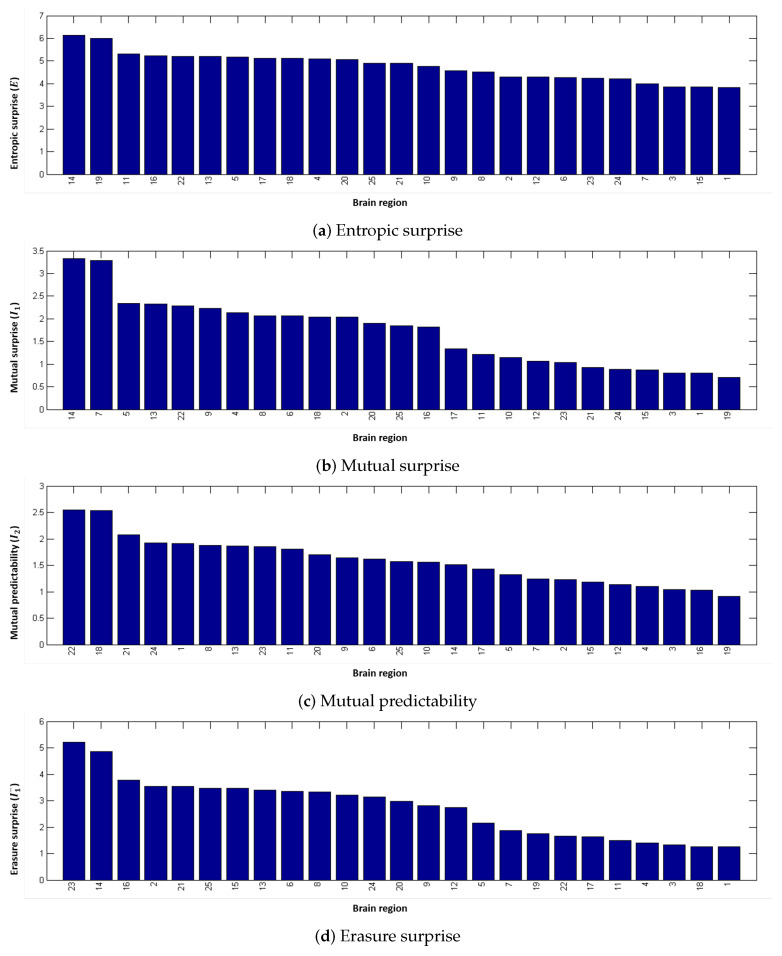
Local measures values ((**a**) entropic surprise; (**b**) mutual surprise (**c**) mutual predictability and (**d**) erasure surprise) obtained with the averaged functional dataset with 25 partitions. An illustrative image of each partition is shown in Figure 15.

**Figure 15:**
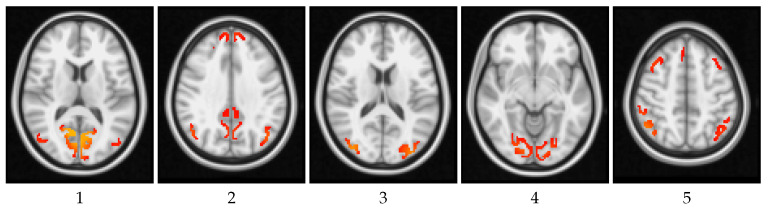

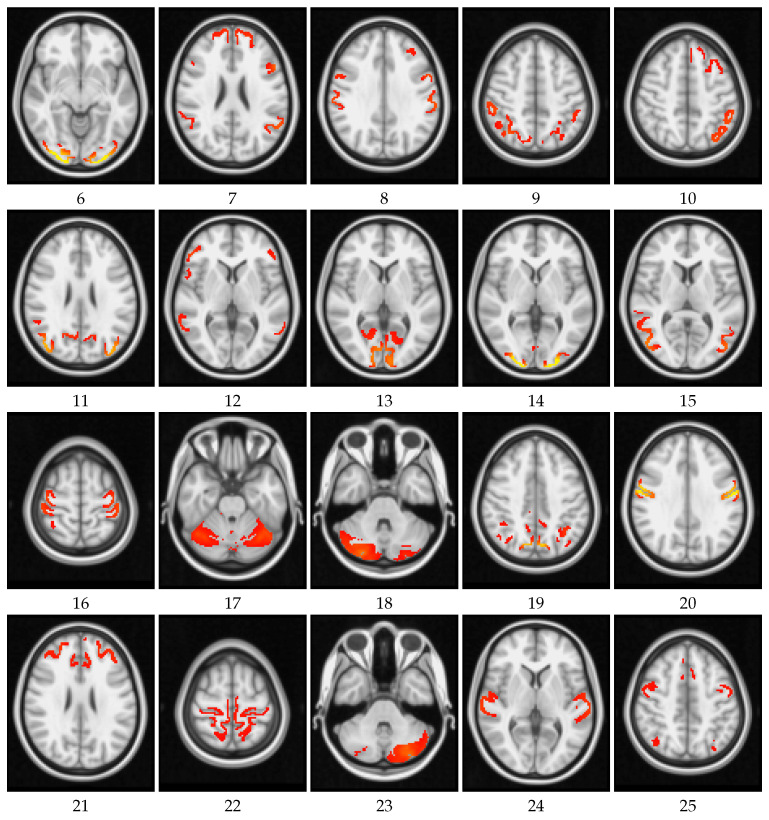
Illustrative images of the 25 regions from the averaged functional dataset [55,56,57].

Figure 14 shows the results for the entropic surprise, the mutual surprise, the mutual predictability, and the erasure surprise applied to the human functional network with 25 partitions. An illustrative image of each partition is shown in Figure 15. Analyzing independently the measures, we found a behavior similar to the structural networks. However, evaluating all the measures and comparing them, we can observe interesting properties. For instance, regions 14 and 19 have both a high erasure surprise value, while mutual surprise is high for region 14 and low for region 19. Thus, these two regions belong to a unique path (due to a high erasure surprise value) but region 14 connects regions highly connected while region 19 connects regions poorly connected (due to the mutual information value). On the other side, region 1, which has a high sum of weights, is also connected to regions similar to itself, so regions with also a big amount of connections. Region 19 has also a low mutual predictability, which means that there is a high capacity to predict the regions which is connected to, on the contrary, region 1 has a lower mutual predictability, so even if it is highly connected to similar nodes it is difficult to predict which are the nodes. Finally, region 1 has a low erasure surprise, which indicates that is likely to belong to a cluster, and region 14 has a high erasure surprise, so it acts more as a bridge of areas which are not strongly connected with other areas.

These results show a proof of principle of the proposed brain model and the suggested set of measures, that provide robust results using structural or functional data. Prior to a further investigation with more clinical data, the proposed approach provides new insights into the brain complexity which may be of interest in studying the functioning of the brain and the connections between regions.

## 5. Conclusions

In this paper, we have used a Markov process-based brain model in which we apply existent and novel information theory-based measures to characterize new properties of complex brain networks. The main contribution of the paper is the proposal of new local and global measures to describe new properties of brain networks in terms of topology and organization, with the main novelty being the definition of erasure mutual information and the erasure surprise. The proposed measures have been tested on synthetic model networks, increasing the number of nodes and the number of edges, and with structural and functional human networks at different scales.

From our experiments and focusing on global measures, we showed that, given a network, the entropy, describes the overall uncertainty of the nodes connectivity. In addition, mutual information, which is a measure of structure, is able to differentiate the topology of network models. Finally, the erasure mutual information, which is a new measure defined by extending the mutual information, describes how unique the paths for a given network are. With this measure, we show an optimal point for small-world networks.

Focusing on local measures, we observed that the entropic surprise, which describes how connected is a node taking into account all the connections in the network, is inversely proportional to the logarithm of the standard strength measure. The mutual surprise, which defines the connectivity of the neighbor nodes for a given node, allows to identify nodes whose nodes have a high connectivity taking into account all connections. The mutual predictability, which given a node, determines the uncertainty associated to a node in predicting the next node, shows that regions with a high clustering tend to be more predictable. Finally, the erasure surprise, which takes into account previous and next nodes, defines how unique the path is which the node belongs to. Results show that regions with a high strength belong to a module where all nodes are strongly connected. The consistency of the results for structural and functional human networks demonstrates the robustness of the proposed measures.

In future work, we will analyze in detail the properties of specific anatomical areas of the human brain and we will study how it can help to detect different diseases. Furthermore, we will investigate clinically informative visualizations using the presented measures.

## Figures and Tables

**Table 1:** Summary of the proposed set of measures.

	Global	Local
**Stationary**	Entropy	Entropic surprise
**Causal**	Mutual Information	Mutual surprise
		Mutual predictability
**Contextual**	Erasure Mutual Information	Erasure surprise
